# Intraocular pressure and cerebral oxygenation during prolonged headward acceleration

**DOI:** 10.1007/s00421-016-3499-3

**Published:** 2016-11-11

**Authors:** Ola Eiken, Michail E. Keramidas, Nigel A. S. Taylor, Mikael Grönkvist

**Affiliations:** 1 Department of Environmental Physiology, Swedish Aerospace Physiology Centre, School of Technology and Health, KTH, Royal Institute of Technology, Berzelius v 13, SE-17165 Stockholm, Sweden; 2 Centre for Human and Applied Physiology, School of Medicine, University of Wollongong, Wollongong, NSW Australia

**Keywords:** Cerebral anoxia reserve, Cerebral blood flow, G tolerance, G-induced loss of consciousness, Oxyhaemoglobin saturation, Retinal anoxia reserve

## Abstract

**Purpose:**

Supra-tolerance head-to-foot directed gravitoinertial load (+Gz) typically induces a sequence of symptoms/signs, including loss of: peripheral vision—central vision—consciousness. The risk of unconsciousness is greater when anti-G-garment failure occurs after prolonged rather than brief exposures, presumably because, in the former condition, mental signs are not consistently preceded by impaired vision. The aims were to investigate if prolonged exposure to moderately elevated +Gz reduces intraocular pressure (IOP; i.e., improves provisions for retinal perfusion), or the cerebral anoxia reserve.

**Methods:**

Subjects were exposed to 4-min +Gz plateaux either at 2 and 3 G (*n* = 10), or at 4 and 5 G (*n* = 12). Measurements included eye-level mean arterial pressure (MAP), oxygenation of the cerebral frontal cortex, and at 2 and 3 G, IOP.

**Results:**

IOP was similar at 1 (14.1 ± 1.6 mmHg), 2 (14.0 ± 1.6 mmHg), and 3 G (14.0 ± 1.6 mmHg). During the G exposures, MAP exhibited an initial prompt drop followed by a partial recovery, end-exposure values being reduced by ≤30 mmHg. Cerebral oxygenation showed a similar initial drop, but without recovery, and was followed by either a plateau or a further slight decrement to a minimum of about −14 μM.

**Conclusion:**

Gz loading did not affect IOP. That cerebral oxygenation remained suppressed throughout these G exposures, despite a concomitant partial recovery of MAP, suggests that the increased risk of unconsciousness upon G-garment failure after prolonged +Gz exposure is due to reduced cerebral anoxia reserve.

## Introduction

A major challenge for pilots flying high-performance aircraft is to maintain adequate retinal and cerebral blood flow during headward acceleration. Thus, high gravitoinertial load in the head-to-seat direction (+Gz; henceforth, G denotes +Gz unless otherwise stated) creates exaggerated intravascular pressure gradients. At 9 G, for instance, heart-level arterial pressure needs to be elevated to 225–275 mmHg to preserve blood perfusion of the brain and retina. The pilots’ arterial pressure is increased by muscular straining manoeuvres in combination with pressurisation of the anti-G suit and, in modern high-performance aircraft, of the breathing gas (Green 2016). Upon a gradual increase in G load, a sequence of symptoms and signs evolves that determines the G-tolerance threshold; the sequence starts with loss of peripheral vision, is followed by loss of central vision and eventually by loss of consciousness (G-LOC). That critical ischaemia is attained at lower G load for the retina than for the cerebral cortex is attributable to two mechanisms. First, to perfuse the retina, arterial pressure at eye level must be sufficient to overcome intraocular pressure (IOP) (Duane 1954; Lambert and Wood 1946; Newsom and Leverett 1968). Second, at increased G load, the “siphone effect” within intracranial vasculature is capable of facilitating perfusion of the cerebral cortex even when local arterial pressure drops to zero or sub-zero levels (Henry et al. 1951), thereby sustaining adequate tissue oxygenation.

To date, the vast majority of studies regarding the influence of excessive G loads on vision and mental performance have been conducted with the G load being elevated from about 1 G to a level exceeding the tolerance threshold. In such conditions, the period of severe retinal or cerebral ischaemia is preceded by normal tissue oxygenation. We have, however, found that the sequence of signs and symptoms upon exposure to a supra-tolerance G load may be affected by the immediate G history preceding the high-G exposure. Thus, experienced fighter pilots were exposed to supra-tolerance G loads either by removing pressure in the G-protective system (anti-G suit and breathing mask) after a prolonged period (2 min) of sustained high-G loading or by rapidly applying such G loading, without pressurising the G-protective system; the pilots were instructed to terminate the G exposure upon loss of peripheral vision. The risk of losing consciousness was several-fold higher when the pressure in the G-protective system was lost after 2 min of high-G loading, than when pressure failed to increase in conjunction with the onset of high G (Eiken and Grönkvist 2013). Presumably, the increased risk of G-LOC in the former condition is due to the fact that after a period of elevated but tolerable G load, warning symptoms in terms of reduced vision do not as distinctly precede G-LOC. This has obvious practical implications, since fighter pilots commonly use the loss of peripheral vision to gauge the tolerable G load. That a prolonged period of high-G load alters the tissue most susceptible to G-induced ischaemia from the peripheral retina to the cerebral cortex may, in turn, be either due to improved provisions for retinal perfusion (i.e., reduced IOP), or to a more pronounced drop of the tissue oxygen reserve in the cerebral cortex than in the retina.

Accordingly, the aims of the present study were to investigate if prolonged exposure to moderately elevated G force reduces either the IOP or the cerebral anoxia reserve. These possibilities were addressed in two separate series of experiments. For practical and safety reasons, IOP was determined at ≤3G (series 1), whereas cerebral oxygenation was predominantly investigated at 4 and 5 G (series 2), because it is known that such G loading may induce substantial hypoxaemia (Barr 1963). Since the anti-G suit, and in particular its abdominal bladder, appears to aggravate the G-induced hypoxaemia (Barr 1963; Eiken et al. 2011), we investigated effects of prolonged G exposures with and without the abdominal bladder included in the suit inflation. Cerebral oxygenation was investigated using a transcranial Near Infrared Spectroscopy (NIRS) technique; the relative drop in frontal cortex oxygenation, as determined by NIRS, has been shown to be a valid risk predictor of G-LOC (Benni et al. 2003; Ryoo et al. 2004; Tripp et al. 2009).

## Methods

**Fig. 1:**
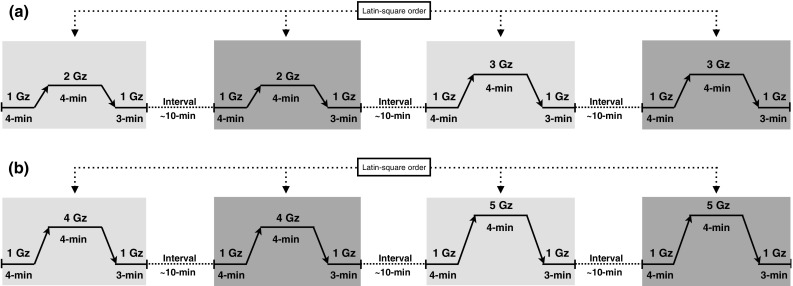
Schematic illustration of the experimental design. The *shaded* zones correspond with the treatment periods (G loading), with the *light shading* referring to complete anti-G-suit (AGS) pressurisation, and the *dark shading* identifying either no AGS pressurisation (**a**), or partial AGS pressurisation (no abdominal bladder; **b**)

Two series of experiments were performed, first, with exposures at 2 and 3 G, addressing the question of changes in IOP and second, with exposures at 4 and 5 G, focussing on the question of cerebral anoxia reserve (Fig. 1). Altogether, 17 healthy individuals (16 men and one woman) participated as subjects, five of whom took part in both experimental series. They gave their written, informed consent prior to enrolling, and were aware that they were free to withdraw from the study at any time. The protocol and experimental procedures were approved by the Regional Human Ethics Committee in Stockholm, Sweden.

The experiments were performed in the 7.25-m radius human-use centrifuge (ASEA, Västerås, Sweden) at the Royal Institute of Technology in Stockholm. During each experiment, the subject was seated in the centrifuge gondola, in a “mock” of the Gripen 39 seat, which has a back angle reclining 28° from the vertical. The centrifuge was controlled by an “open-loop” system, employing pre-set G-time profiles. Gz was measured using an analogue accelerometer mounted in the gondola at a position approximately corresponding to the vertical level of the subjects’ heart. Throughout each experiment, the subject was monitored via closed-circuit television, and was able to communicate with the experimenter by means of a two-way intercom system.

The anti-G ensemble used in the Gripen 39 aircraft (AGE 39) has been described in detail elsewhere (Eiken et al. 2007, 2011). It comprises a pressure-breathing system and an extended coverage pneumatic anti-G suit, which contains a single-compartment inflatable bladder, fully covering the lower abdomen as well as the legs, from below the gluteal region and the inguinal ligaments to the ankles. For the purpose of the present experiments, a modified version of the anti-G suit was used, in which it was possible to pressurise the abdominal and leg portions of the bladder separately (cf. Eiken et al. 2011). As in the aircraft, pressurisation of the anti-G suit commenced at 2 G, with pressure increasing linearly with increasing G load to reach a maximum of 19.1 kPa (143 mmHg) at 5.0 G; at G loads ≤2 G, the suit was supplied with a “ready pressure” of 1.3 kPa. Pressurisation of the airways commenced at 4 G, with a stipulated pressure of 1.34 kPa at 5 G. AGE pressures were controlled through a G valve/breathing regulator (Eros, F-5341, Eros, Plaisir Cedex, France).

## Experimental protocols

### Series 1

Ten subjects (nine men and one woman; mean ± SD age: 36 ± 14 years, height: 181 ± 6 cm, body mass: 74 ± 13 kg) participated. On a separate occasion, preceding the experimental day, each subject was trained in performing the IOP measurements in the centrifuge gondola at 1 G (stationary), using the procedure described below. On the experimental day, the subject was instrumented and then positioned in the gondola wearing the anti-G suit. He/she was investigated in four conditions: 2 G, once with the full AGS (2-G AGS) and once without the AGS pressurised (2-G NoAGS), and at 3 G with (3-G AGS) and without pressurised AGS (3-G NoAGS). The order of these conditions was alternated and balanced among subjects in a Latin-square manner. Each experimental trial started with 4 min of baseline measurements at 1 G. Thereafter, the G load was increased by 0.5 G/s to a plateau of either 2 or 3 G. After 4 min at the plateau, the load was decreased to 1 G by 0.5 G/s, and measurements continued for another 3 min. Successive trials were interspersed by at least a 10-min recovery period.

### Series 2

Twelve male subjects (age: 30 ± 10 years, height: 183 ± 4 cm, body mass: 81 ± 7 kg) participated. Each subject was instrumented and equipped with the complete AGE 39. He was investigated in four conditions: 4 Gz, once with the full AGS (including the abdominal bladder) pressurised (4-G ABD) and once without the abdominal bladder pressurised (4-G NoABD), and at 5 G with and without the abdominal bladder pressurised (5-G ABD and 5-G NoABD, respectively). Note that in the 5-G exposures the breathing gas was also pressurised (see above). The order of the conditions was similarly alternated among subjects in a balanced fashion. Each G exposure started with 4 min of baseline measurements at 1 G, followed by a G-load increase (0.5 G/s) to a 4-min G plateau of 4 or 5 G. After 4 min, the load was decreased to 1 G by 0.5 G/s, and measurements continued for another 3 min. The G exposures were interspersed by at least 10 min of recovery.

## Physiological measurements

*Heart rate* (*HR*) *and arterial pressure* HR was derived from electrocardiographic recordings using a cardiometer (Datex-Engström, Instrumentation Corp, Helsinki, Finland), and with the electrodes positioned in a precordial 5-lead arrangement. Systolic (SAP), diastolic (DAP) and mean (MAP) arterial pressures were measured using a volume-clamp technique (Portapres, TNO, Amsterdam, The Netherlands) with the pressure cuff placed around the middle-phalanx of the third finger of the right hand, and with the reference pressure transducer taped to the skin of the temple, at the level of the eyes. The right arm was supported by an armrest adjusted, so that the distal portions of the fingers were at a vertical level corresponding to the *jugulum sterni*.

*Tonometry* IOP was measured using a non-contact tonometer (Pulsair, Intellpuff, Keeler, UK), that was mounted on a custom-made horizontal slide, positioned in the centrifuge gondola with the tonometer transducer at the vertical and transversal levels of the subject’s left eye. The subject could move the tonometer in the sagittal direction, along a frictionless track; the tonometer was spring-loaded, so that it retracted away from the face when the subject released his/her grip of it. The tonometer was provided with a video camera (Plexgear USB Microscope) and a miniature video screen (7 inch TFT LCD Monitor), enabling the subject, using his/her right eye, to place the tonometer in the correct position for IOP measurement of his/her left eye. IOP readings were monitored via a standard CCD video camera. The subject was instructed to conduct as many IOP measurements as possible during the course of each intervention. Typically, due to nystagmus, IOP measurements could not be performed during, and for a few seconds following, each G transition.

*Capillary oxyhaemoglobin saturation* (*SpO*_*2*_) SpO_2_ was measured continuously throughout all trials using a pulse oxymeter (Nellcor Puritan Bennett Inc., Pleasanton, CA, USA), with the transducer placed on the second finger of the right hand. The oxymeter has an accuracy of ±2% units across the range 70–100% and an acceptable resilience to motion artefacts.

*Cerebral oxygenation* Throughout each experiment, the oxygenation of the frontal cerebral cortex was measured using continuous-wave NIRS (NIRO-200NX, Hamamatsu, Japan). The transducer unit was positioned over the left prefrontal cortex between the first frontal-polar (Fp1) and the third frontal (F3) locations, as determined using the modified international 10–20 system for electroencephalograms. To minimise confounding influence of skin blood flow, the unit, which consists of an emitter and a detector, was taped to the skin at a fix inter-optode distance of 4.0 cm (Hampson and Piantadosi 1988). To reduce intrusion of external light and loss of transmitted NIR light from the measuring area, the transducer unit was covered with an opaque bandage.

The NIR light is comprised of three wavelengths (735, 810 and 850 nm), and changes in tissue oxygenation were calculated as oxygenated (∆[O_2_Hb]) and deoxygenated (∆[HHb]) haemoglobin (Hb), respectively. In addition, total Hb (∆[THb]), which is the sum of ∆[O_2_Hb] and ∆[HHb], and reflects changes in regional blood volume (Van Beekvelt et al. 2001), was calculated continuously. The theory, limitations, and reliability of cerebral oxygenation obtained employing NIRS have been reviewed elsewhere (Boushel et al. 2001; Ferrari et al. 2004). The NIRS signal was recorded at 5 Hz and expressed relative to the 4-min baseline period preceding each trial.

## Analyses

Data obtained during and after the G plateaux were averaged every 15 s. However, because of large inter-individual variability regarding the number of IOP measurements during the G exposures, IOP values were averaged for each min within subjects.

Statistical analyses were performed using Statistica 8.0 (StatSoft, Tulsa, OK, USA). All data are reported as mean ± SD, unless otherwise indicated. Analysis of the normal distribution of the data was performed with the Kolmogorov–Smirnov test. The statistical significance of differences was evaluated by a two-way (condition × time) general linear model repeated measures analysis of variance (ANOVA). Mauchly’s test was conducted to assess the sphericity, and the Greenhouse-Geisser correction was used to adjust the degrees of freedom when the assumption of sphericity was not satisfied. The Tukey honestly significant difference post hoc test was employed to identify specific differences between means when ANOVA revealed a significant F ratio for interaction or main effects. Probabilities <0.05 were regarded as being statistically significant.

## Results

All experiments were conducted without adverse events; none of the subjects experienced G-LOC.

### Series 1

**Fig. 2:**
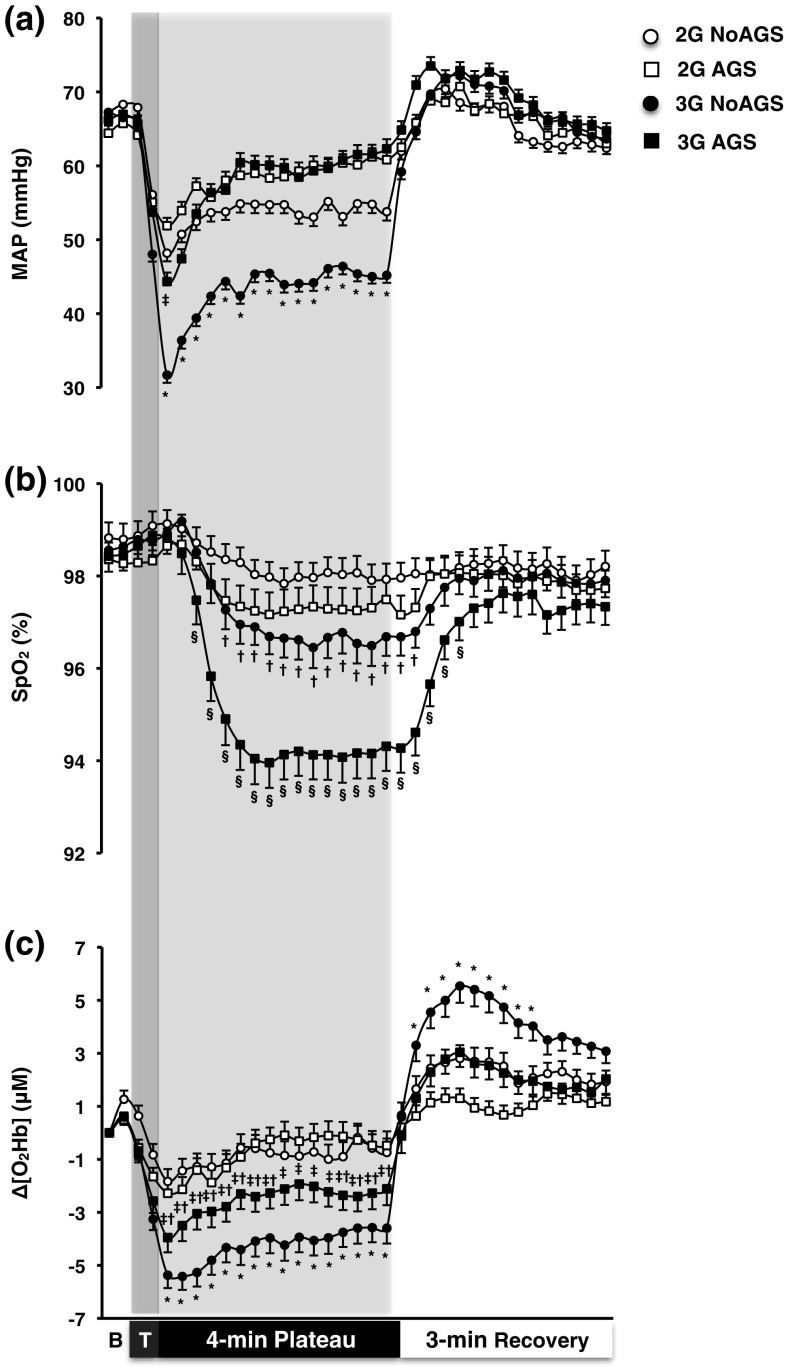
Mean (SE) arterial pressure (MAP; **a**), capillary oxyhaemoglobin saturation (SpO_2_; **b**) and changes from resting values in cerebral oxyhaemoglobin (Δ[O_2_Hb]; **c**) obtained during the 4-min G plateaux at 2 and 3 G, and the 3-min recovery with (AGS) and without (NoAGS) anti-G-suit pressurised. *B* baseline phase, *T* transition (G onset) phase. *Significantly different from 2-G NoAGS, 2-G AGS, and 3-G AGS, ^†^significantly different from 2-G NoAGS, ^‡^significantly different from 2-G AGS, ^§^significantly different from 2-G NoAGS, 2-G AGC, and 3-G NoAGS (*p* ≤ 0.05). *n* = 10

*Arterial pressure and heart rate* In all trials, MAP dropped instantly upon G exposure, reaching a nadir value ~15 s into the G plateau (Fig. 2a). That drop was followed by a gradual increase with a complete recovery (*p* > 0.05) to baseline MAP during both trials conducted with the AGS pressurised (after ~120 s at 2 G and ~75 s at 3-G AGS). Without suit pressurisation (NoAGS trials), only a partial recovery was observed (*p* < 0.05). Throughout the G plateau, MAP was consistently lower in the 3-G NoAGS than in the other trials (*p* < 0.001). Upon returning to 1 G, MAP promptly reverted to baseline values in all trials (*p* > 0.05).

During the G exposure, SAP decreased in both NoAGS trials, but remained unchanged in the AGS trials (Table 1). Each G exposure reduced DAP and elevated HR (*p* < 0.05), with changes being more pronounced in the 3-G NoAGS than in the other trials (*p* < 0.01; Table 1).

**Table 1:** Mean (± SD) values of systolic (SAP) and diastolic (DAP) arterial pressure, heart rate (HR), and changes in cerebral frontal cortex deoxyhaemoglobin (Δ[HHb]) and total haemoglobin (Δ[THb]) obtained during the 4-min baseline (B), the 4-min plateau at 2 and 3 G, and the 3-min recovery (R), with (AGS) and without (NoAGS) the anti-G-suit pressurised

	2-G NoAGS			2-G AGS			3-G NoAGS			3-G AGS		
	*B*	G plateau	*R*	*B*	G plateau	*R*	*B*	G plateau	*R*	*B*	G plateau	*R*
SAP (mmHg)	127 ± 22	114 ± 29*	122 ± 27	119 ± 18	114 ± 24	122 ± 22	125 ± 18	107 ± 29*	123 ± 26	125 ± 23	124 ± 26†	126 ± 24
DAP (mmHg)	46 ± 9	33 ± 11*	46 ± 8	45 ± 8	35 ± 9*	46 ± 8	46 ± 5	22 ± 9*^‡^	47 ± 7	45 ± 8	34 ± 11*	49 ± 10
HR (beats min^−1^)	72 ± 12	86 ± 12*^#^	75 ± 14	69 ± 11	79 ± 13*^††^	73 ± 13	71 ± 11	103 ± 13*^§^	79 ± 16	71 ± 12	89 ± 11*	77 ± 15
Δ[HHb] (μM)	0	−1.96 ± 3.10	0.76 ± 1.83	0	−0.58 ± 1.91	−0.30 ± 1.18	0	−0.20 ± 2.89	2.44 ± 2.88	0	−0.64 ± 2.94	1.26 ± 1.83
Δ[THb] (μM)	0	−3.51 ± 4.10*	2.49 ± 3.57	0	−1.62 ± 3.55	0.47 ± 1.91	0	−4.77 ± 5.04*^§^	6.11 ± 5.23	0	−3.48 ± 5.28*	3.06 ± 4.45

*n* = 10

* Significantly different from the baseline and recovery

^†^Significantly different from 2-G NoAGS, 2-G AGS, and 3-G NoAGS

^‡^Significantly different from 2-G NoAGS, 2-G AGS, and 3-G AGS

^§^Significantly different from 3-G AGG

^#^Significantly different from 2-G AGS and 3-G NoAGS

^††^Significantly different from 3-G NoAGS and 3-G AGS

*Capillary oxyhaemoglobin saturation* SpO_2_ remained unchanged during the 2-G NoAGS trial (*p* > 0.05; Fig. 2b). During the 3-G AGS trial, SpO_2_ dropped and levelled off within ~75 s; whereas in the 2-G AGS and 3-G NoAGS trials, SpO_2_ decreased to a stable level within ~90 s (*p* < 0.05). Following those G exposures, SpO_2_ gradually recovered in a trial-dependent manner, such that it had resumed baseline values within ~30, ~45, and ~90 s in the 2-G AGS, 3-G NoAGS, and 3-G AGS trials, respectively. During the G plateau and the initial part of recovery, the reduction in SpO_2_ was substantially greater in the 3-G AGS than in the other trials (*p* ≤ 0.01). SpO_2_ was also lower in the 3-G NoAGS than in the 2-G NoAGS trial (*p* = 0.02).

*Cerebral oxygenation* In all trials, Δ[O_2_Hb] dropped instantly upon G exposure, reaching a nadir value ~15 s into the plateau period (*p* < 0.01; Fig. 2c). During the remaining course of the G plateau, Δ[O_2_Hb] did not exhibit any further significant change in any of the trials (*p* > 0.05). Throughout the G plateau, Δ[O_2_Hb] was consistently lower in the 3-G NoAGS than in the other trials (*p* < 0.05), and lower in the 3-G AGS than in both 2-G trials (*p* ≤ 0.05). Upon return to 1 G, Δ[O_2_Hb] rebounded promptly; during the recovery phase, Δ[O_2_Hb] was greater in the 3-G NoAGS trial than in the other trials (*p* ≤ 0.05).

During the G exposure, Δ[HHb] did not change in any of the trials (*p* > 0.05; Table 1). Δ[THb] decreased by ~135, ~303. and ~261% in 2-G NoAGS, 3-G NoAGS, and 3-G AGS trials, respectively (*p* < 0.05; Table 1). Δ[THb] was reduced by ~134% in the 2-G AGS trial, although that difference was not statistically significant (*p* = 0.88). The drop in Δ[THb] was greater in the 3-G NoAGS than in the 3-G AGS trial (*p* < 0.001).

*Intraocular pressure* No statistically significant difference in IOP was detected between 1-G baseline periods and the 4-min periods at the 2- and 3-G plateaux (*p* = 0.09; Table 2). No difference was observed between the trials (*p* = 0.68; Table 2).

**Table 2:** Mean values ± SD as well as (total number of measurements) of intraocular pressure obtained during the 4-min baseline, the 4-min plateau at 2 and 3 G, and the 3-min recovery period, with (AGS) and without (NoAGS) the anti-G-suit pressurised

	Baseline	G plateau				Recovery
		1 min	2 min	3 min	4 min	
2-G NoAGS	14.1 ± 1.6 (291)	14.1 ± 1.6 (61)	14.1 ± 1.57 (89)	14.0 ± 1.6 (91)	14.0 ± 1.6 (94)	14.2 ± 1.6 (239)
2-G AGS	14.1 ± 1.6 (320)	14.1 ± 1.6 (52)	14.0 ± 1.56 (74)	14.0 ± 1.6 (67)	14.1 ± 1.6 (101)	14.2 ± 1.6 (210)
3-G NoAGS	14.1 ± 1.6 (362)	14.1 ± 1.6 (36)	14.1 ± 1.58 (70)	14.1 ± 1.6 (65)	14.0 ± 1.6 (80)	14.1 ± 1.6 (220)
3-G AGS	14.1 ± 1.7 (308)	14.1 ± 1.7 (51)	14.1 ± 1.66 (84)	14.1 ± 1.6 (69)	14.1 ± 1.6 (69)	14.1 ± 1.7 (207)

*n* = 10

### Series 2

**Fig. 3:**
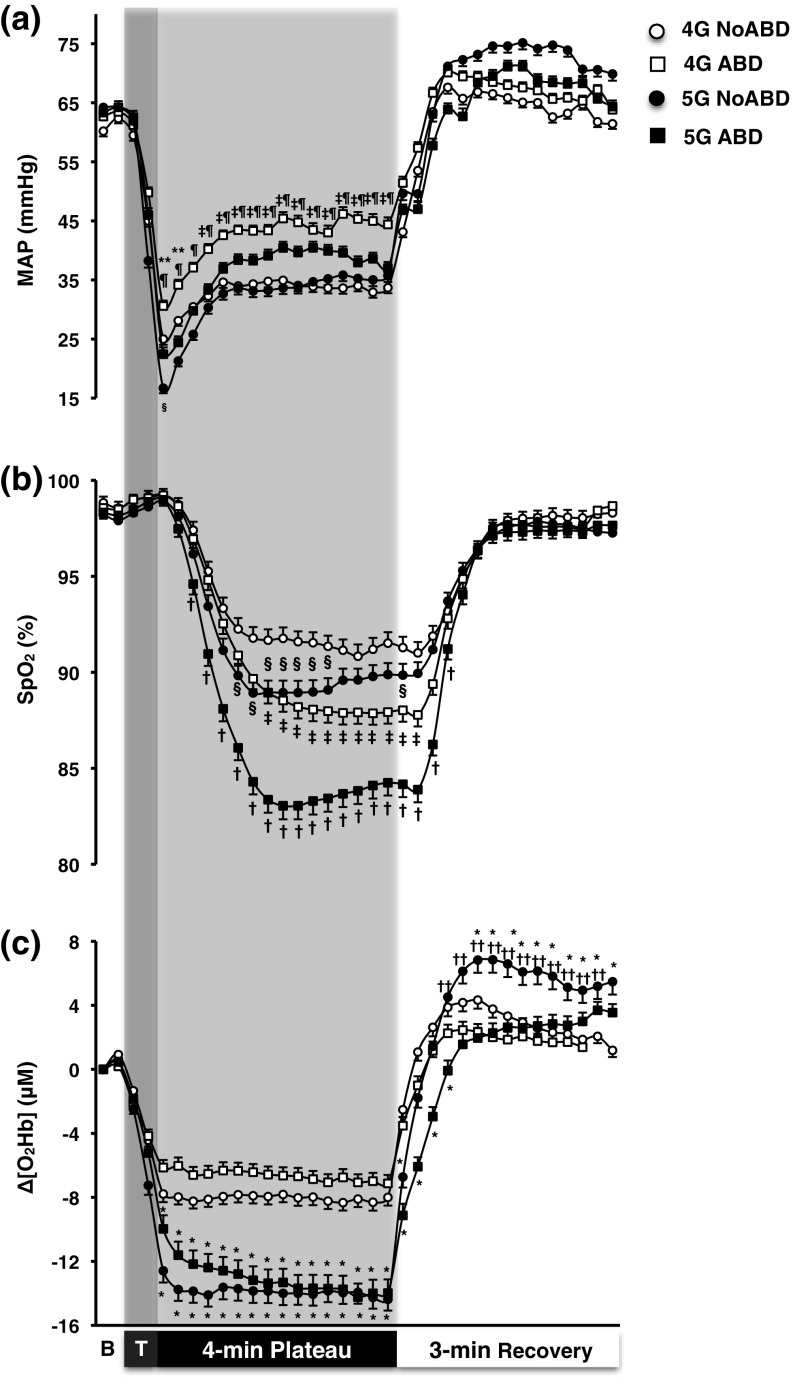
Mean (SE) arterial pressure (MAP; **a**), capillary oxyhaemoglobin saturation (SpO_2_; **b**) and changes from resting values in cerebral oxyhaemoglobin (Δ[O_2_Hb]; **c**) obtained during the 4-min G plateaux at 4 and 5 G, and the 3-min recovery, with (ABD) and without (NoABD) the abdominal bladder of the anti-G-suit inflated. *B* baseline phase, *T* transition (G onset) phase. *Significantly different from 4-G NoABD and 4-G ABD, ^†^significantly different from 4-G NoABD, 4-G ABD, and 5-G NoABD, ^‡^significant difference between 4-G NoABD and 4-G ABD, ^§^significant difference between 4-G NoABD and 5-G NoABD, ^¶^significant difference between 4-G ABD and 5-G NoABD, **significant difference between 4-G ABD and 5-G ABD, ^††^significant difference between 5-G NoABD and 5-G ABD; (*p* ≤ 0.05). *n* = 12

*Arterial pressure and heart rate* In all trials, MAP dropped instantly upon G exposure, reaching a nadir value ~15 s into the G plateau phase (*p* < 0.001; Fig. 3a). Thereafter, MAP gradually recovered, especially in the ABD trials, but remained below baseline values throughout the G plateau (*p* < 0.001). MAP was higher in the 4-G ABD than in either of the other trials (*p* < 0.05). It was also slightly higher in the 5-G ABD than in the 5-G NoABD trial, although that difference was not significant (*p* = 0.61). During the recovery phase, MAP reverted to baseline values within ~45 s, and no differences were observed between the trials (*p* > 0.05).

During the G exposures, SAP and DAP were substantially reduced (*p* ≤ 0.05); no differences were observed between the trials (*p* > 0.05; Table 3). HR was elevated in all trials, and that increase was greater in the 5-G than the 4-G trials (*p* ≤ 0.001; Table 3).

**Table 3:** Mean (±SD) values of systolic (SAP) and diastolic (DAP) arterial pressure, heart rate (HR), and changes in cerebral frontal cortex deoxyhaemoglobin (Δ[HHb]) and total haemoglobin (Δ[THb]) obtained during the 4-min baseline (B), the 4-min plateau at 4 and 5 G, and the 3-min recovery (R), with (ABD) and without (NoABD) the abdominal bladder of the anti-G-suit inflated

	4-G NoABD			4-G ABD			5-G NoABD			5-G ABD		
	*B*	G plateau	*R*	*B*	G plateau	*R*	*B*	G plateau	*R*	*B*	G plateau	*R*
SAP (mmHg)	123 ± 17	105 ± 19*	124 ± 23	121 ± 13	109 ± 23*	123 ± 21	121 ± 16	106 ± 31*	127 ± 23	121 ± 20	103 ± 23*	120 ± 18
DAP (mmHg)	42 ± 8	14 ± 10*	44 ± 10	44 ± 8	24 ± 11*	48 ± 9	46 ± 12	17 ± 25*	52(15)	45 ± 11	16 ± 13*	47 ± 13
HR (beats·min^−1^)	81 ± 13	110 ± 14*	88 ± 11	80 ± 14	109 ± 15*	87 ± 13	81 ± 10	119 ± 16*^†^	96 ± 12	83 ± 11	119 ± 14*^†^	93 ± 11
Δ[HHb] (μM)	0	1.11 ± 2.75	3.04 ± 2.66	0	1.21 ± 2.77	1.72 ± 2.72	0	1.58 ± 5.48	5.31 ± 7.03	0	0.87 ± 6.43	0.99 ± 2.98
Δ[THb] (μM)	0	−8.05 ± 4.52*	5.37 ± 5.52	0	−7.12 ± 4.66*	2.96 ± 5.64	0	−15.23 ± 10.95*^†^	10.04 ± 14.14	0	−15.46 ± 11.04*^†^	1.50 ± 6.55

*n* = 12

* Significantly different from the baseline and recovery

^†^Significantly different from 2G-NoAGS, 2G-AGS, and 3G-NoAGS

*Capillary oxyhaemoglobin saturation* In all trials, SpO_2_ dropped and levelled off within ~60 s of high-G onset (*p* < 0.001), and recovered within ~90 s upon returning to 1 G (Fig. 3b). SpO_2_ was lower in the 5-G NoABD than in the 4-G NoABD trial (*p* = 0.02). Pressurisation of the AGS abdominal bladder aggravated the G-induced reduction in SpO_2_, with SpO_2_ being substantially lower in the 5-G ABD than in the other trials (*p* < 0.001). It was also lower in the 4-G ABD than in the 4-G NoABD and 5-G NoABD trials (*p* < 0.01).

*Cerebral oxygenation* In all trials, Δ[O_2_Hb] dropped promptly upon each G exposure (*p* < 0.001; Fig. 3c), and the reduction was more pronounced at 5 G than at 4 G (*p* < 0.001). During both 4-G trials and also during the 5-G NoABD trial, Δ[O_2_Hb] dropped and levelled off within 15 s of the G onset. In the 5-G ABD trial, however, the initial drop in Δ[O_2_Hb] was followed by a further gradual decrease with the nadir value being attained at the end of the G exposure. Upon return to 1 G, Δ[O_2_Hb] rebounded within ~30 s in all trials; yet, in the 5-G ABD trial, the recovery of Δ[O_2_Hb] was slightly slower than in the 4-G trials (*p* < 0.05). During the recovery phase, Δ[O_2_Hb] was higher following 5-G NoABD than following the other trials (*p* < 0.05).

During these G exposures, Δ[HHb] did not change in any of the trials (*p* > 0.05; Table 3). Δ[THb] was substantially reduced in all trials (*p* < 0.001), and especially within the 5-G trials (*p* < 0.01; Table 3).

## Discussion

Present results demonstrated that IOP was but minimally reduced by prolonged exposures to moderately elevated gravitoinertial load in the head-to-foot direction. By contrast, each high-G exposure induced a prompt reduction in cerebral oxygenation, the magnitude of which was dependent both on the size of the step-change in G load and on whether, and what kind of, G-protective garment the subject was using. In the 4- and 5-G exposures, the initial depression of cerebral oxygenation prevailed at a steady level, or oxygenation even tended to drop further during the course of the 4-min G plateaux. This response occurred despite the fact that the initial decline in local arterial pressure was followed by a partial recovery during the latter two-thirds of the plateau.

These results should be viewed in the context of our previous study (Eiken and Grönkvist 2013), which showed that the risks of near and actual loss of consciousness are several-fold greater if pressure in the anti-G system is lost after a 2-min period of sustained high-G load than if the system fails to elevate pressure during onset of such G loading, presumably because in the former condition loss of consciousness is not always preceded by impeded vision. This raises the question of whether prolonged exposure to elevated but tolerable G loads either reduces the risk of G-induced loss of vision, or increases the risk of G-induced cerebral dysfunction.

### Intraocular pressure at increased G load

Judging from our finding that the IOP was virtually unaffected by a threefold increase in G load, it appears unlikely that prolonged exposure to high, but tolerable, G loads might reduce the susceptibility to decrements in peripheral and central vision upon exposure to supra-tolerance loads. Thus, it has long been recognised that the G-induced loss of peripheral and foveal visions, commonly referred to by pilots as “grey out” and “black out”, respectively, are caused by retinal ischaemia resulting from insufficient perfusion pressure in intraocular blood vessels (Duane 1954; Lambert and Wood 1946; Newsom and Leverett 1968); once MAP drops below IOP, retinal perfusion ceases (Riva et al. 1986).

A sustained reduction of arterial pressure may, in turn, reduce IOP (Mitchell et al. 2005), but the magnitude of any IOP drop induced by a sustained decrease in arterial pressure, will amount to a mere fraction of the actual drop in arterial pressure (Mitchell et al. 2005; Nicholson et al. 1968). Consequently, the only conceivable period during which an MAP-induced drop in IOP might predispose to increased ocular perfusion pressure, is the transient period between regaining MAP and the ensuing readjustment of the IOP to the elevated MAP. At the outset of the present experiments, we reasoned that improved provisions for retinal blood flow might occur during prolonged G exposure, either by way of a transient overshoot in retinal perfusion pressure following the recovery of MAP after its transient initial drop, or by another unforeseen mechanism. It appears, however, that IOP is rather resilient to G-induced decrements in local arterial pressure. Thus, even at 3 G without pressurised anti-G suit, IOP exhibited a minimal drop, despite MAP stabilising at about 20 mmHg below the baseline (1-G) level. Whether the G-induced drops in MAP were of sufficient magnitude and duration to significantly affect IOP remains to be established.

Bakke et al. (2009) showed that a 2-min isometric exercise bout resulting in an MAP increase from 80 to 120 mmHg was paralleled by a 4-mmHg increase in IOP, whereas a decrease in local MAP of 20–25 mmHg accompanying a postural change from recumbent to sitting results in a rapid decrease in IOP (Krieglstein and Langham 1975), that may vary considerably in magnitude, with average changes reported in different studies ranging from 0.3 to 2.9 mm Hg (cf. Anderson and Grant 1973; Jain and Marmion 1976; Krieglstein and Langham 1975). In addition, long-term changes in arterial pressure appear to result in IOP changes of similar magnitude, with a 1-mmHg change in IOP for every 30-mmHg change in SAP (Mitchell et al. 2005). The reason for the large inter-study variations in IOP response to a 20–25 mmHg drop in local arterial pressure remains to be settled, as does the reason for the minute IOP change in response to a 20-mmHg drop in local MAP in the present 3-G exposures. The non-contact tonometry technique used in the present study is regarded a reproducible and sensitive means of measuring IOP (Almubrad and Ogbuehi 2010; Jain and Marmion 1976).

### Cerebral oxygenation at increased G load

The second main aim of the present experiments was to investigate the effects of sustained moderate elevations of G load on the cerebral anoxia reserve. Oxygen delivery to the frontal cortex is determined by the local blood flow and the arterial oxygen content. As expected, all sustained G-load increments induced an immediate drop in eye-level MAP, which then partially recovered, but nevertheless stabilised considerably below its 1-G baseline value, during the latter two-thirds of the G plateaux. By contrast, frontal cortex oxygenation (Δ[O_2_Hb]) decreased promptly upon high-G exposure and did not recover during the ensuing course of the G plateau; during the 5-G exposure, with pressurisation of the full AGS, including the abdominal bladder, the initial drop in frontal cortex oxygenation was even followed by a slight but further drop in oxygenation. That Δ[O_2_Hb] remained reduced throughout the G exposure may be attributed to two mechanisms, reduced local blood flow and hypoxaemia.

In the 4- and 5-G trials, the steady-state eye-level MAP varied between 30 and 45 mmHg and was dependent not only on the G load but also of whether the G-suit abdominal bladder was pressurised, with a distinctly higher MAP at a given G load, when the bladder was pressurised. Cerebral blood flow is predominantly autoregulated, vascular myogenic tone being governed by a complex interplay between responses to local changes in transmural pressure and the chemical environment, but also being modified by regional vascular conducted responses (cf Jensen and Holstein-Rathlou 2013). Particularly in dynamic conditions, the interplay between autoregulatory mechanisms is not fully understood (cf. Panerai et al. 2002). Regardless, it is clear that the cerebral vasculature regulates flow across a wide range of perfusion pressures, whereas under 1-G conditions, its autoregulatory capacity is exceeded once local arterial pressure drops below about 60 mmHg (Testart 1990). Thus, it is reasonable to assume that in the present 4- and 5-G exposures, cerebral blood flow remained reduced throughout the high-G plateaux.

The reduction in cerebral blood flow resulting from the G-induced drop in local arterial pressure might have been modulated by two mechanisms, acting in opposite directions. On the one hand, at increased G loads, cerebral perfusion pressure is influenced by the siphon effect (Henry et al. 1951). Since intracranially, venous walls are not as collapsible as in other regions of the body, local venous pressure may assume subatmospheric levels during headward acceleration. Hence, antegrade flow may prevail in cerebral vasculature even in the face of a G-induced drop in local arterial pressure to or below 0 mmHg (Henry et al. 1951). Even though the existence of the siphon effect is well established (Green 2016), it is difficult to quantify its role at different G loads, since data of intracerebral venous pressures in humans exposed to such conditions are scarce. On the other hand, it can be presumed that the G-induced hypoxaemia led to hyperpnoea and therefore to hypocapnia. Although hypocapnia constitutes a potent stimulus for cerebral vasoconstriction (Shapiro et al. 1970), it appears unlikely that it was capable of overriding the vasodilatory effect of the G-induced drop in local precapillary transmural pressure. Thus, the prompt overshoot in frontal cortex oxygenation upon cessation of G exposure probably reflected a concomitant surge in cerebral blood flow and hence suggests that local precapillary resistance vessels were dilated upon release of the G load. Similar overshoot responses in Δ[O_2_Hb], reflecting a post-ischaemic reactive hyperaemia phase, have been noted following cessation of short-duration high +Gz loads (Kobayashi et al. 2012) and in particular following exposures inducing G-LOC (Ryoo et al. 2004).

The present G-induced hypoxaemia is attributable to pulmonary atelectasis and consequent shunting of deoxygenised blood in dependent portions of the lungs (Barr 1962, 1963; Glaister 1970). Thus, the G-dependent intrathoracic hydrostatic pressure gradient results in basal redistribution of the intrapulmonary blood volume and compression of basal alveoli (Barr 1963; Dussault et al. 2016; Glaister 1970). Our finding that the G-induced hypoxaemia was exaggerated by inflation of the AGS abdominal bladder is also in agreement with results from previous studies (Barr 1963) and supports the notion that G-induced hydrostatic compression atelectasis is aggravated by transmission of pressure from the AGS to the abdominal cavity and the lower thorax (Eiken et al. 2011). It should be noted that the magnitude and time courses of the present G-induced hypoxaemia episodes, as reflected by the decrements in capillary oxygen haemoglobin saturation in a fingertip, should be interpreted with caution. As obvious from the SpO_2_ curve upon G offset, changes in oxyhaemoglobin saturation induced in the pulmonary circulation will appear in the fingertip with a delay of at least 30 s. In the cerebral vessels, the delay time is substantially shorter, and, in all likelihood, the magnitude of the saturation drop is larger than in the capillaries of a finger (cf. Lindholm et al. 2007).

It appears that in the present 4- and 5-G exposures (but not in the 2- and 3-G exposures), the hypoxaemia was sufficiently severe to counteract any recovery of cerebral deoxygenation despite the considerable recovery of cerebral perfusion pressure during the latter part of the exposure. In fact, in the condition most relevant from an operational viewpoint, namely 5 G with full anti-G garment, ∆[O_2_Hb] continued to drop by about 40% during the course of the G load (from −10 to −14 μM). Judging from the considerable difference in ∆[O_2_Hb] between the 4- and 5-G ABD trials, it seems reasonable to assume that the continuous drop in ∆[O_2_Hb] during the course of the G plateau might be more pronounced at loads exceeding 5 G.

Thus, progressive reduction of the cerebral anoxia reserve during the exposure to sustained high G whilst wearing full anti-G-protective garment remains a plausible explanation to our previous finding that the risk of G-LOC was several-fold higher when the pressure in the G-protective system was lost after a prolonged period of sustained exposure to +6 Gz than when pressure failed to increase in conjunction with the onset of +6 Gz (Eiken and Grönkvist 2013). In this connection, it should be noted that the retinal anoxia reserve, by contrast, appears rather resilient to hypoxaemia. Thus, a preceding period of moderate hypoxia does not seem to affect the latency period from complete blocking of retinal blood flow to loss of vision (Lambert and Bjurstedt 1962).

Information is scarce regarding oxygenation responses of the frontal cortex to prolonged periods of sustained high-G loads. In the present 5-G exposures, ∆[O_2_Hb] dropped by about 14 μM, which should be compared to the 20–23 μM decrements reported in conjunction with brief +Gz exposures resulting in G-LOC (Kurihara et al. 2007; Tripp et al. 2009). It should be noted, however, that although ∆[O_2_Hb] appears to be a valid predictor of A-LOC and G-LOC (Kurihara et al. 2007; Ryoo et al. 2004; Tripp et al. 2009), the relative drop in ∆[O_2_Hb] at which A-LOC/G-LOC occurs varies considerably between studies [e.g., from −6 to −7% in the study by Ryoo et al. (2004) to −32 to −33% in the study by Kurihara et al. (2007)]. Presumably, these inter-study variations are mainly attributable to methodological differences, including spectrometer performance, inter-optode distance, and transducer position. Regardless, inter-study comparisons of ∆[O_2_Hb] in response to G exposure should be conducted with caution.

### Study limitations and delimitations

**Fig. 4:**
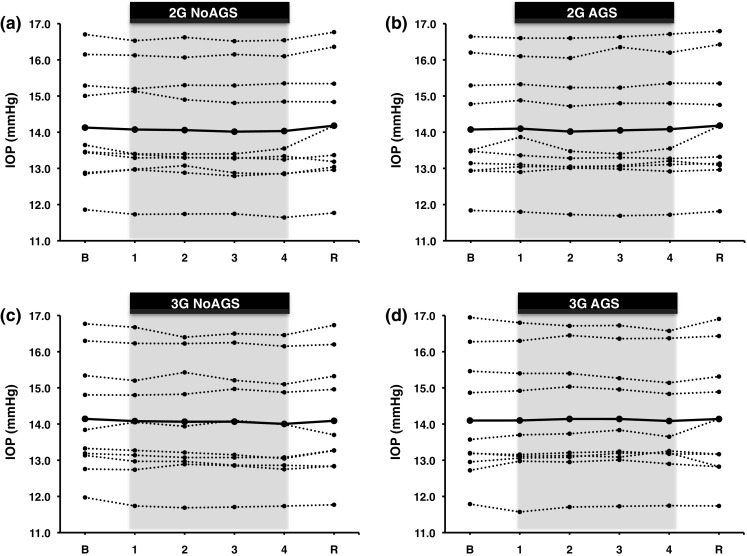
Individual mean values, and group averages (*bold curves*), of intraocular pressure (IOP) obtained during the 4-min G plateaux at 2 and 3 G, and the 3-min recovery with (AGS) and without (NoAGS) anti-G-suit pressurised. *B* baseline phase, *R* recovery phase. *n* = 10

Arguably, the present study lacked sufficient statistical power to rule out the possibility that increased +Gz loading may reduce IOP. From a practical viewpoint, it appears, however, that any IOP change induced by slight-to-moderate +Gz elevations is too small to have significant consequences (Fig. 4). Present G exposures consisted of 4-min plateaux at ≤ +5 Gz. From an operational viewpoint, it is important that these data are supplemented with data from G exposures comprising simulated aerial combat manoeuvres (SACM), during which the G loading is alternated (e.g., every 15 s) between moderate (e.g., 4.5 G) and high (e.g., 7 G). Judging from previous studies, long-duration SACM will induce similar levels of hypoxaemia (Balldin and Siegborn 1992), and hence presumably also of frontal cortex hypoxia, as those observed in the present long-duration, constant-load G exposures. In-flight recordings from a few F-15 pilots seem to suggest that, during ACM, cerebral oxygenation decreases in response to both the peak load and duration of the ACM (Kobayashi et al. 2002). Consequently, it is reasonable to assume, but remains to be established, that currently observed gradually diminishing cerebral oxygenation at elevated G load might also occur during long-duration SACM. Furthermore, it remains to be investigated whether, and in what manner, pressure breathing, G load, and G-suit pressurisation interact as regard both intrapulmonary shunting of deoxygenized blood and cerebral oxygenation. In the present study, the increase in G load from 4 to 5 G was accompanied not only by an increase in anti-G-suit pressure, but also by application of positive pressure breathing.

## Conclusions

Prolonged exposure to light-moderate +Gz elevation did not affect intraocular pressure but induced a sustained reduction in cerebral oxygenation, despite a concomitant partial recovery of mean arterial pressure. That cerebral oxygenation remained suppressed throughout these exposures is attributable to pulmonary shunting of deoxygenized blood into the systemic arteries, and suggests that the increased risk of G-LOC upon G-garment failure after prolonged G exposure is due to a reduction in the cerebral anoxia reserve.

## Abbreviations

ABD: With the full anti-G suit (including the abdominal bladder) pressurised
AGE 39: Anti-G ensemble used in the Gripen 39 aircraft
AGS: Anti-G suit
A-LOC: Almost G-induced loss of consciousness
DAP: Diastolic arterial pressure
∆[Hb]: Change in deoxygenated haemoglobin
∆[O_2_Hb]: Change in oxygenated haemoglobin
∆[THb]: Change in total haemoglobin
G: Dimensionless quantity denoting the ratio between the vector sum of gravitational and inertial forces and Earths gravity
G-LOC: G-induced loss of consciousness
+Gz: Gravitoinertial force in the head-to-foot direction
Hb: Haemoglobin
HR: Heart rate
IOP: Intraocular pressure
MAP: Mean arterial pressure
NIRS: Near infrared spectroscopy
NoABD: With the anti-G suit (excluding the abdominal bladder) pressurised
NoAGS: Without the anti-G suit
SACM: Simulated aerial combat manoeuvres
SAP: Systolic arterial pressure
SpO_2_: Capillary oxyhaemoglobin saturation
